# Evaluating Tissue-Agnostic Approvals in Thoracic and Head and Neck Malignancies

**DOI:** 10.3390/cancers18050856

**Published:** 2026-03-06

**Authors:** Daniel Thomas Jones, Rishi Kumar Nanda, Abbas Ali Hussain, Riccesha Hattin, Yin Mon Myat, Rajat Thawani, Jeremy Cetnar, Mohamed Shanshal, Kyaw Zin Thein, Shivaani Kummar

**Affiliations:** 1 Department of Internal Medicine, Sunrise Health GME Consortium, Las Vegas, NV 89148, USA; 2 College of Osteopathic Medicine, Touro University Nevada, Las Vegas, NV 89014, USA; rnanda24@gmail.com; 3 Department of Internal Medicine, Kirk Kerkorian School of Medicine at University of Nevada, Las Vegas (UNLV), Las Vegas, NV 89106, USA; abbasalihussain7@gmail.com (A.A.H.); rhattin20@gmail.com (R.H.); 4 Department of Internal Medicine, Interfaith Medical Center, Brooklyn, NY 11213, USA; yinmon.myat@obhny.org; 5 Division of Hematology and Medical Oncology, Knight Cancer Institute, Oregon Health & Science University (OHSU), Portland, OR 97239, USA; thawani@ohsu.edu (R.T.); cetnarj@ohsu.edu (J.C.); kummar@ohsu.edu (S.K.); 6 Division of Hematology and Oncology, Vanderbilt University Medical Center, Nashville, TN 37232, USA; mohamed.shanshal@vumc.org; 7 Division of Hematology and Medical Oncology, Comprehensive Cancer Centers of Nevada, Las Vegas, NV 89169, USA; kyaw.thein@usoncology.com

**Keywords:** tumor-agnostic therapy, tissue-agnostic oncology, thoracic cancer, head and neck cancer, biomarker-driven therapy, next-generation sequencing, MSI-H/dMMR, TMB-High, NTRK fusion, RET fusion, BRAF V600E, HER2 IHC 3+

## Abstract

Background/Objectives: Tissue-agnostic therapy has transformed oncology by enabling treatment selection based on molecular alterations rather than tumor origin. Since 2017, nine U.S. Food and Drug Administration approvals across six biomarker classes have defined this paradigm. Thoracic and head and neck (H&N) cancers have been underrepresented in the registrational evidence supporting these approvals. This review systematically evaluated biomarker representation, histologic distribution, and clinical applicability of tissue-agnostic therapies in thoracic and H&N malignancies. Methods: A narrative systematic review was conducted using PubMed, ClinicalTrials.gov, and regulatory documents for all tissue-agnostic approvals between January 2017 and October 2025. Data were extracted from pivotal trials, including total enrollment, objective response rate (ORR), histologic distribution, and thoracic/H&N representation. Emerging biomarkers and resistance mechanisms were assessed from phase I–III studies and basket trials. Results: Nine tissue-agnostic approvals encompassing six biomarkers were identified: MSI-H/dMMR, TMB-High, NTRK, RET, BRAF V600E, and HER2 (IHC 3+). Across pivotal datasets (3800 patients), thoracic and H&N cancers accounted for fewer than 8% (*n* = 290) of enrolled patients. Thoracic representation was dominated by non-small-cell lung cancer (NSCLC) in RET, NTRK, and HER2 programs (150 patients, 4%), while small-cell lung, mesothelioma, and thymic carcinomas contributed <1% combined. H&N cancers comprised 140 patients (3–4%), primarily secretory salivary carcinoma in NTRK trials (n = 12–20), thyroid carcinoma in BRAF (*n* = 36) and RET (*n* = 45) programs, and rare HER2-positive salivary duct carcinomas. Conventional HNSCC and sinonasal cancers were limited to 1–2 cases per trial. Only two of nine trials (22%) reported prespecified CNS endpoints, and RNA-based fusion testing was employed in <40%, underscoring diagnostic variability and limited applicability. Conclusions: Although tissue-agnostic therapy has expanded the reach of precision oncology, thoracic and H&N cancers remain underrepresented in registrational evidence. Most approvals rely on single-arm basket studies with small, heterogeneous subsets that preclude histology-specific conclusions. Future research should prioritize histology-enriched trial designs, standardized molecular diagnostics, and real-world validation to establish reliable, equitable standards of care for these underrepresented malignancies.

## Simple Summary

This review examines how recent cancer drug approvals have shifted from organ-specific indications to biomarker-defined, or “tissue-agnostic,” approaches. These therapies target molecular alterations that drive tumorigenesis across histologies, including NTRK fusions treated with larotrectinib, entrectinib, and repotrectinib; RET fusions responsive to selpercatinib; BRAF V600E mutations targeted with the combination of dabrafenib and trametinib; HER2 overexpression (IHC 3+) treated with fam-trastuzumab deruxtecan-nxki; microsatellite instability-high (MSI-H) or mismatch repair deficiency (dMMR) tumors treated with pembrolizumab or dostarlimab; and high-tumor-mutational-burden (TMB-H) tumors eligible for pembrolizumab. Despite these advances, thoracic and head and neck cancers have been inconsistently represented in the pivotal trials supporting such approvals. This review synthesizes evidence from all nine U.S. Food and Drug Administration tissue-agnostic approvals, critically evaluates their applicability to thoracic and head and neck malignancies, and highlights emerging biomarkers and ongoing challenges related to molecular testing, therapeutic resistance, and equitable access. These insights aim to inform trial design and enhance the implementation of precision oncology in underrepresented tumor types.

## 1. Introduction

Thoracic and head and neck (H&N) malignancies constitute a biologically diverse group of tumors characterized by distinct molecular profiles and clinical behaviors. Despite advances in surgery, radiation, and systemic therapy, outcomes remain poor for most advanced-stage diseases. Conventional cytotoxic regimens rarely achieve durable responses, underscoring the need for strategies guided by molecular rather than anatomic classification [1]. The integration of next-generation sequencing (NGS) into routine oncology has enabled systematic identification of oncogenic drivers and accelerated the transition toward biomarker-based precision medicine.

Since 2017, the United States Food and Drug Administration (FDA) has granted nine tissue-agnostic approvals across six biomarker classes: microsatellite instability-high or deficient mismatch repair (MSI-H/dMMR), tumor mutational burden-high (TMB-High), neurotrophic tyrosine receptor kinase (NTRK) fusions, rearranged-during-transfection (RET) fusions, BRAF V600E mutations, and HER2 immunohistochemistry 3+ overexpression. These approvals, spanning immune checkpoint inhibitors, kinase inhibitors, and antibody–drug conjugates, have established the feasibility of treating cancer based on shared molecular alterations rather than tissue of origin.

Thoracic and H&N malignancies have contributed unevenly to the registrational evidence supporting these approvals. Strong efficacy signals have been observed in subsets such as RET-rearranged non-small-cell lung and thyroid carcinomas, NTRK fusion-positive secretory salivary carcinoma, and BRAF V600E-mutated anaplastic thyroid carcinoma, while other histologies remain underrepresented. This review synthesizes current evidence on tissue-agnostic therapies in thoracic and H&N cancers, identifies existing evidence gaps, and outlines priorities necessary to optimize biomarker-driven care in these underrepresented malignancies.

Several recent reviews have summarized tissue-agnostic approvals broadly, including conceptual frameworks for histology-agnostic versus histology-modulated actionability and overviews of approved and emerging biomarkers. However, these prior syntheses are typically pan-cancer in emphasis and do not quantify, using registrational datasets, the degree to which thoracic and head and neck malignancies are represented, nor do they consistently map those denominators to practical issues that matter in thoracic/H&N clinics (fusion-testing modality, CNS endpoint reporting, and histology-specific interpretability). The novelty of the present review is threefold: (i) we systematically aggregate the pivotal evidence underlying all nine FDA tissue-agnostic approvals and quantify thoracic/H&N representation across programs, (ii) we interpret these data through a thoracic/H&N lens (including CNS relevance and diagnostic platform limitations), and (iii) we outline trial-design and testing priorities needed to generate actionable, equitable evidence for these historically underrepresented tumor types.

## 2. Conceptual and Regulatory Foundations of Tissue-Agnostic Therapy

### 2.1. Literature Search Strategy and Study Selection

This review was conducted using a structured literature search of PubMed, Embase, and FDA regulatory databases through January 2026. Search terms included combinations of “tumor-agnostic,” “tissue-agnostic,” “histology-agnostic,” “basket trial,” “FDA approval,” and the names of individual targeted agents associated with tumor-agnostic indications. We additionally cross-referenced FDA approval summaries, prescribing information documents, and pivotal trial publications to ensure completeness and regulatory accuracy.

Inclusion criteria consisted of FDA-approved tumor-agnostic therapies supported by prospective clinical trial data, with extraction of enrollment numbers, histology-specific representation, and reported efficacy outcomes (ORR, DoR, PFS, OS when available). Studies limited to histology-specific approvals were excluded unless referenced for contextual comparison.

Data extraction was performed manually from primary trial publications and FDA review documents. Because this manuscript synthesizes regulatory approvals and pivotal trial characteristics rather than performing a pooled meta-analysis, a formal PRISMA flow diagram was not applied. Structured search and verification methods were used to ensure transparency and reproducibility.

### 2.2. From Organ-Specific to Biomarker-Driven Oncology

Historically, cancers were classified according to their anatomic origin and histologic features such as morphology, differentiation, and tissue architecture. This framework reliably correlated with prognosis and therapeutic strategy but often failed to capture the molecular heterogeneity within morphologically similar tumors. Patients with identical histologies frequently exhibited divergent treatment responses, reflecting biological diversity that histology alone could not explain [2,3].

The advent of molecular diagnostics and next-generation sequencing (NGS) redefined this approach by enabling tumor classification based on genetic and transcriptomic alterations, tumor mutational burden, and immune signatures. Tumor-agnostic therapy refers to treatment determined by a shared biomarker rather than the site of origin [4]. Oncogenic drivers such as NTRK fusions, RET fusions, BRAF V600E mutations, HER2 overexpression (IHC 3+), mismatch repair deficiency, and high tumor mutational burden occur across multiple histologies and can predict response to targeted or immune-based therapies.

This biomarker-driven paradigm complements, rather than replaces, histologic classification by adding molecular precision to traditional pathology. It expands therapeutic access for rare malignancies, enhances treatment personalization by matching therapy to a tumor’s dominant oncogenic dependency rather than its anatomic label, and can accelerate drug development. In practice, “personalization” means that two patients with different primary sites may receive the same targeted agent when they share a high-confidence driver alteration (e.g., TRK inhibition for NTRK fusions in secretory salivary carcinoma or NSCLC; RET inhibition for RET fusions in lung and selected non-lung tumors), while patients with the same histology may appropriately receive different systemic therapy when their biomarkers diverge. This biomarker-first alignment can improve the probability of response, avoid ineffective empiric regimens, and enable earlier use of CNS-active targeted agents in tumor types with brain-metastasis risk [3,4].

### 2.3. Trial Designs Driving Tumor-Agnostic Approvals

The development of tumor-agnostic therapies has been enabled by innovative trial methodologies, particularly basket trials. A basket trial enrolls patients with different tumor histologies that share a single molecular alteration (the “basket” is defined by the biomarker), and evaluates whether that alteration predicts benefit across lineages. This differs from an umbrella trial, which enrolls a single cancer type (one histology) and assigns patients to multiple biomarker-defined sub-studies, and from platform trials, which use an adaptive, multi-arm infrastructure that can add or drop therapies over time. The key advantage of basket designs is feasibility: rare genomic subsets can be studied without requiring separate histology-specific trials, enabling earlier signal detection and supporting regulatory decisions when randomized trials are impractical [5]. By grouping patients according to biomarker status rather than tissue of origin, basket trials efficiently assess whether a molecular alteration predicts therapeutic benefit across malignancies. Operationally, patients are grouped after a prespecified test identifies eligibility, NGS detects an NTRK fusion, IHC shows HER2 IHC 3+, or a validated assay reports TMB ≥ 10 mut/Mb. All eligible patients are then analyzed within the biomarker-defined cohort (and, ideally, with prespecified histology subgroups). For example, an NTRK basket cohort may include secretory salivary carcinoma, thyroid carcinoma, and NSCLC; efficacy can be reported for the overall biomarker cohort and secondarily for each histology to reveal lineage-specific differences that may be masked in pooled estimates.

Because randomized controlled trials are often not feasible in small biomarker-defined populations, basket studies typically use single-arm designs that rely on surrogate efficacy endpoints such as objective response rate and duration of response. These measures provide early evidence of clinical activity and have served as the evidentiary basis for multiple regulatory approvals [6].

The absence of control arms limits comparative interpretation, and biological heterogeneity across tumor lineages may influence outcomes even when the same biomarker is present. Despite these limitations, basket trials have provided robust proof-of-concept data supporting histology-independent efficacy and established the regulatory foundation for modern tissue-agnostic approvals, redefining how precision oncology trials are designed and evaluated [2].

### 2.4. Regulatory Pathways and Innovations

Tumor-agnostic approvals have been enabled by regulatory mechanisms designed for high-need settings and small biomarker-defined populations. A central pathway is accelerated approval, which can rely on surrogate endpoints such as objective response rate (ORR) and duration of response (DoR) when randomized trials or overall survival analyses are impractical in rare subsets. In parallel, breakthrough therapy designation and priority review can compress development timelines when early evidence suggests substantial improvement over available therapy, and orphan drug designation can support programs in rare molecular subsets [5].

Companion diagnostics have become integral to tumor-agnostic labeling because eligibility hinges on standardized biomarker detection. For example, tumor mutational burden-high approval frameworks specify validated assays (and assay-specific thresholds), while fusion-driven indications depend on reliable detection of oncogenic rearrangements. Importantly, the regulatory logic is biomarker-centric, but the evidentiary strength can remain histology-modulated: when certain tumor lineages dominate enrollment (as often occurs), pooled efficacy may not translate uniformly to underrepresented cancers. These realities motivate (i) prespecified histology reporting, (ii) harmonization of testing methods across sites, and (iii) confirmatory strategies using real-world evidence or histology-enriched cohorts to ensure that tumor-agnostic labels remain clinically meaningful across diverse tumor types [2].

### 2.5. Current Limitations and Challenges

Although tumor-agnostic approvals have broadened precision oncology, multiple challenges constrain their clinical application. A major limitation is the underrepresentation of many cancer types in registrational datasets. Because biomarker-defined populations are rare, basket trials often pool diverse histologies, improving feasibility but obscuring lineage-specific differences in efficacy [6].

Reliance on single-arm designs and surrogate endpoints such as objective response rate and duration of response provides proof-of-concept activity but does not establish durable benefit or comparative effectiveness against standard treatments [5]. The lack of prospective histology-stratified analyses further limits interpretation of outcomes.

Diagnostic variability compounds these issues. Differences in sequencing platforms, biomarker thresholds, and companion diagnostic assays create inconsistencies in identifying eligible patients, while inequitable access to comprehensive molecular testing risks widening disparities [4].

These limitations highlight the need for refined trial design and diagnostic harmonization. Future studies should incorporate histology-enriched cohorts, standardized biomarker assays, and outcome reporting stratified by tumor type to ensure reliable and equitable implementation of tumor-agnostic therapies.

## 3. Evolution and Regulatory Milestones of Tumor-Agnostic Approvals

The development of tumor-agnostic therapy was enabled by basket and multi-cohort trials that pooled biomarker-defined cancers across histologies, demonstrating that oncogenic drivers can predict treatment response independent of tissue of origin and paving the way for regulatory precedents.

In 2017, pembrolizumab received the first histology-independent FDA approval for MSI-H/dMMR tumors. The decision drew on pooled KEYNOTE datasets that demonstrated durable PD-1 responses across multiple tumor types, including the initial biomarker-selected proof of concept in KEYNOTE-016, activity in non-colorectal MSI-H/dMMR tumors in KEYNOTE-158, and confirmation in treatment-refractory metastatic colorectal cancer in KEYNOTE-164. These studies showed substantial and durable response rates across 15 tumor types, establishing MSI-H/dMMR as a cross-histology predictor of PD-1 benefit [7,8,9,10,11,12].

In 2018, the selective TRK inhibitor larotrectinib was approved for NTRK fusion-positive tumors based on an integrated analysis across LOXO-TRK-14001, SCOUT, and NAVIGATE. Adults and children with 17 tumor types were included, with a blinded independent review overall response rate of 75%, complete responses in 13%, and the majority of responses ongoing at data cutoff. SCOUT contributed pediatric safety and antitumor activity, while NAVIGATE provided the largest multicenter basket experience to support histology-independent efficacy [13,14].

In 2019, entrectinib was approved for NTRK fusion-positive tumors, supported by an integrated efficacy set from ALKA-372-001, STARTRK-1, and STARTRK-2. The pooled adult analysis reported an overall response rate of 57% with a median duration of response of 10 months and median progression-free survival of 11 months. Entrectinib demonstrated clinically meaningful intracranial activity, with an intracranial overall response rate of 55% among patients who had brain metastases at baseline, reinforcing its relevance in tumor types with high CNS involvement [15,16,17].

In 2020, pembrolizumab received a second tissue-agnostic indication for tumors with high tumor mutational burden, defined as at least 10 mutations per megabase. This approval was based on a prespecified exploratory biomarker analysis of KEYNOTE-158 that showed an overall response rate of 29% in the TMB-High subgroup, with responses that were often durable. FoundationOne companion diagnostic (CDx) assay was specified as the companion diagnostic for identifying TMB-High status in the label [8,17].

In 2021, dostarlimab earned accelerated approval for recurrent or advanced dMMR/MSI-H solid tumors. The multicohort, single-arm GARNET study demonstrated a clinically meaningful overall response rate of 44% with 10% complete responses in the dMMR endometrial cohort and durable benefit at the time of analysis. A non-endometrial dMMR cohort provided supportive cross-histology evidence for the tumor-agnostic label [18].

In 2022, selpercatinib became the first tumor-agnostic RET fusion therapy. The LIBRETTO-001 basket cohort of non-lung and non-thyroid RET fusion-positive tumors showed an overall response rate of 44% with a median duration of response of 24 months and a median progression-free survival of 13 months, supporting durable activity across multiple histologies beyond NSCLC and thyroid carcinoma [19,20].

Also in 2022, the combination of dabrafenib plus trametinib was approved for BRAF V600E-mutated solid tumors. The phase 2 ROAR basket trial reported robust efficacy in anaplastic thyroid carcinoma with an overall response rate of 56%, a median duration of response 14 months, and a median overall survival of 14 to 15 months. The NCI-MATCH Subprotocol H provided additional cross-histology support, including responses in non-thyroid cohorts such as lung adenocarcinoma [21,22,23].

In 2024, repotrectinib received accelerated approval for NTRK fusion-positive tumors. TRIDENT-1 included TKI-naïve and TKI-pretreated NTRK cohorts and demonstrated meaningful activity, including in NSCLC subsets, with confirmed response rates of 62% in TKI-naïve disease and 42% after prior TRK inhibitor exposure. The program also characterized central nervous system penetration at the recommended dose, a clinically relevant attribute for tumors with high CNS tropism [24].

Later in 2024, fam-trastuzumab deruxtecan achieved tissue-agnostic approval for HER2-positive (IHC 3+) tumors. DESTINY-PanTumor02 reported an overall response rate of 37% with a median duration of response of 11 months, median progression-free survival of 7 months, and median overall survival of 13 months across multiple histologies. Thoracic relevance was strengthened by HER2-overexpressing NSCLC cohorts in DESTINY-Lung01, and DESTINY-CRC02 provided additional confirmatory activity in colorectal cancer [25,26,27]. The chronology of these tumor-agnostic FDA approvals, their associated biomarkers, and pivotal supporting trials is summarized in Table 1.

## 4. Molecular Landscape and Diagnostic Challenges in Thoracic and Head & Neck Cancers

The molecular epidemiology of thoracic and head and neck (H&N) cancers explains their limited representation in tissue-agnostic approvals. Biomarkers such as RET, NTRK, BRAF, HER2, MSI-H/dMMR, and TMB-High occur at low and variable frequencies in these tumors [7,10,19,28]. This rarity has led to underrepresentation in registrational datasets, with most evidence derived from other organ systems [20].

Diagnostic limitations further constrain inclusion. DNA-based sequencing can miss complex or rare gene fusions better identified with RNA-based assays, yet RNA testing remains underutilized [29]. This limitation is especially relevant in thoracic and H&N oncology because several actionable alterations in these diseases are fusion-driven and may involve complex breakpoints or low-expression transcripts that are more reliably detected by RNA-based assays. In lung cancer, clinically important fusions (including rare partners and atypical breakpoints) can be missed by DNA-only panels, particularly when intronic regions are poorly covered; missed fusions directly translate into missed opportunities for CNS-active targeted therapy. In head and neck cancers, fusion-heavy subtypes such as secretory salivary carcinoma (classically NTRK fusion-positive) and selected salivary duct/sinonasal entities may be under-identified when RNA testing is not performed, compounding the already low real-world testing penetration in many H&N settings. Accordingly, a thoracic/H&N “testing-first” strategy often requires paired DNA + RNA sequencing (or reflex RNA fusion testing when DNA is negative but suspicion remains) to minimize false negatives in precisely the tumor types most dependent on accurate fusion identification. Tumor mutational burden (TMB) assessment also varies widely across platforms, as differences in panel size, algorithms, and cutoffs yield inconsistent classifications [8,17]. Even minor methodological variation can alter biomarker status, fragmenting already small cohorts.

Beyond assay-level limitations, real-world testing penetration itself represents a critical diagnostic challenge, particularly in head and neck squamous cell carcinoma (HNSCC). In a large U.S. claims-based analysis of 63,209 adults with metastatic solid tumors, next-generation sequencing (NGS) utilization differed markedly by tumor type. There were 18% of patients with metastatic non-small-cell lung cancer (NSCLC) underwent NGS in the post-2018 era, compared with only 2% of patients with metastatic head and neck cancer [30]. Although histologic subtypes were not separately specified, head and neck cancer populations in claims-based datasets are predominantly composed of squamous cell carcinoma, making these findings highly relevant to conventional HNSCC. These data indicate that limited testing uptake, rather than biomarker rarity alone, substantially restricts identification of actionable alterations in head and neck cancers.

Technical factors such as tumor heterogeneity, low cellularity, and degraded formalin-fixed samples contribute to false negatives. Access to comprehensive NGS is inconsistent outside tertiary centers, reducing detection of rare but actionable alterations [19]. Liquid biopsy has emerging utility in NSCLC but remains unvalidated in H&N cancers.

Interpretation is further influenced by lineage-specific biology. In NSCLC, co-mutations such as STK11 and KEAP1 attenuate TMB’s predictive value, while in HNSCC, human papillomavirus (HPV) status modulates immunotherapy responsiveness [8,17].

Without standardized biomarker assays and broader RNA-based fusion testing, evidence for thoracic and H&N cancers will remain heterogeneous. These diagnostic and molecular limitations explain their persistent underrepresentation in pivotal tissue-agnostic studies.

The application of tumor-agnostic approvals in thoracic and head and neck oncology requires careful clinical judgment when disease-specific representation in registrational trials is limited. In scenarios where thoracic or H&N enrollment comprised a small fraction of study populations, clinicians must interpret pooled response rates cautiously and consider biologic plausibility, CNS penetration data, and molecular testing reliability before extrapolating benefit.

When evidence is sparse, shared decision-making becomes particularly important, incorporating factors such as performance status, prior therapies, alternative approved options, and the strength of biomarker-target dependency. In thoracic malignancies, where brain metastases are common, CNS activity may meaningfully influence treatment choice even when histology-specific enrollment is limited. In head and neck cancers, careful molecular confirmation is critical given historically low testing penetration in certain practice settings.

An additional limitation relates to small subgroup sizes and wide confidence intervals within thoracic and head and neck cohorts across multiple registrational studies. In several approvals, thoracic or H&N representation comprised only a minority of enrolled patients, limiting statistical power for histology-specific inference. Wide confidence intervals around subgroup ORR estimates reduce precision and increase uncertainty when extrapolating pooled efficacy to underrepresented cancers. Prior methodological analyses have highlighted the risk of overinterpretation of small biomarker-defined subgroups in basket trials, particularly when heterogeneity across histologies may exist. These considerations underscore the importance of cautious interpretation and the need for confirmatory data in lineage-specific settings.

Tumor-agnostic approvals should be viewed as biologically rational therapeutic opportunities that require histology-informed contextualization rather than automatic generalization.

## 5. Evidence Base by Biomarker: Tumor-Agnostic Approvals

The regulatory foundation for tumor-agnostic therapy now includes nine FDA approvals across six biomarker categories. These are pembrolizumab (2017) and dostarlimab (2021) for MSI-H/dMMR tumors [7,10,18], pembrolizumab (2020) for TMB-High tumors [8,17], the TRK inhibitors larotrectinib (2018), entrectinib (2019), and repotrectinib (2024) for NTRK fusions [13,16,31], dabrafenib plus trametinib (2022) for BRAF V600E-mutated tumors [21,22], selpercatinib (2022) for RET fusions [19,20], and fam-trastuzumab deruxtecan (T-DxD, 2024) for HER2-positive (IHC 3+) tumors [25,26,27]. Each approval was supported by basket or multi-cohort studies that demonstrated clinically meaningful activity across multiple histologies.

Despite these achievements, the evidentiary depth remains uneven in thoracic and head and neck cancers. Stronger data exist for NSCLC, thyroid carcinoma, secretory salivary carcinoma, and salivary duct carcinoma, while other thoracic and H&N histologies remain underrepresented or absent from the decision-enabling datasets. As a result, important questions persist regarding response durability, intracranial activity, and long-term safety in these underrepresented diseases.

The subsections below summarize the trial designs, efficacy outcomes, and safety signals for each biomarker class, with particular attention to thoracic and head and neck cancers to clarify where the evidence is sufficient to inform practice and where gaps remain that warrant prospective, histology-enriched enrollment.

### 5.1. NTRK Fusions: Larotrectinib

Larotrectinib was the first selective TRK inhibitor to receive FDA tumor-agnostic approval in 2018, marking a major advance in precision oncology by demonstrating approval based on a molecular alteration rather than histology. The approval was supported by an integrated analysis of 55 patients across three early-phase trials (LOXO-TRK-14001, SCOUT, NAVIGATE) that included 17 tumor types. The overall response rate (ORR) was 75% (95% CI, 61–85) with 13% complete responses. At a median follow-up of 9.4 months, 71% of responses were ongoing and 55% of patients remained progression-free. Median duration of response (DoR) and progression-free survival (PFS) were not reached [13].

Secretory salivary carcinoma accounted for 12 patients (22%), thyroid carcinoma for 5 (9%), and lung tumors for 4 (7%). No small-cell lung, mesothelioma, thymic, or sinonasal cancers were included. Subgroup outcomes such as ORR, DoR, and PFS were not reported by histology, and only 1 patient (2%) had baseline brain metastases, limiting assessment of central nervous system activity.

In the SCOUT pediatric trial, only 2 children with papillary thyroid carcinoma were enrolled; both remained on therapy beyond seven months without progression [14]. Most thoracic and head and neck cases were captured within NAVIGATE, but efficacy outcomes were pooled across tumor types, leaving histology-specific results unresolved.

The rarity of NTRK fusions in thoracic cancers (<1% in NSCLC) and in most thyroid carcinomas outside secretory subtypes explains this underrepresentation. Secretory salivary carcinoma, where NTRK fusions are nearly universal, provided the strongest evidence for approval. Diagnostic variability further limits real-world applicability, as DNA-based sequencing can miss rare or complex fusions, and RNA-based next-generation sequencing, though more sensitive, is not routinely available.

Larotrectinib established the regulatory foundation for tumor-agnostic therapy but highlighted the minimal inclusion of thoracic and head and neck malignancies. While secretory salivary carcinoma demonstrated clear efficacy, evidence for thyroid carcinoma and NSCLC remains limited, and no data exist for small-cell, thymic, mesothelioma, or sinonasal primaries [13,14].

### 5.2. NTRK Fusions: Entrectinib

Entrectinib, a multikinase inhibitor targeting TRKA, TRKB, TRKC, ROS1, and ALK, received FDA tumor-agnostic approval in 2019. The approval was based on an integrated analysis of 54 adults with NTRK fusion-positive solid tumors from three trials: ALKA-372-001, STARTRK-1, and STARTRK-2. Pediatric data from STARTRK-NG were included only in safety analyses. By blinded independent review, the overall response rate (ORR) was 57% (95% CI, 43–71) with 7% complete responses. The median duration of response (DoR) was 10.4 months (95% CI, 7.1–not estimable), median progression-free survival (PFS) was 11.2 months (95% CI, 8.0–14.9), and median overall survival (OS) was 21.0 months (95% CI, 14.9–not estimable) [16].

Thoracic and head and neck representation remained limited. Non-small-cell lung cancer (NSCLC) comprised 10 patients (19%) with an ORR of 70%, secretory salivary carcinoma accounted for 7 patients (13%) with 6 confirmed responses (86%), and thyroid carcinoma included 5 patients (9%) with 1 responder (20%). No small-cell lung cancer, mesothelioma, thymic carcinoma, or sinonasal primaries were enrolled [16]. This imbalance reflects the rarity of NTRK fusions across thoracic histologies and their near-pathognomonic presence in secretory salivary carcinoma.

A distinctive feature of entrectinib was its demonstrated intracranial activity. Among 12 patients (22%) with baseline brain metastases, the intracranial ORR was 55% (95% CI, 23–83), with a median intracranial PFS of 14 months and median time to CNS progression of 17 months [16]. These findings are particularly relevant for thoracic cancers such as NSCLC, where brain metastases are frequent and CNS-active targeted therapies are clinically important.

Most efficacy data originated from STARTRK-2, which contributed 94% (51 of 54) of patients, while ALKA-372-001 and STARTRK-1 added only three. The earlier trials primarily informed safety and pharmacokinetics, and histology-specific outcomes were not estimable at the individual trial level [15,16].

Entrectinib reinforced the tumor-agnostic paradigm by confirming that TRK inhibition produces durable responses across histologies and by demonstrating meaningful CNS penetration. Thoracic and head and neck malignancies, particularly small-cell, mesothelioma, thymic, and sinonasal cancers, were underrepresented. The strongest signal remains in secretory salivary carcinoma, while thyroid carcinoma and NSCLC data are numerically limited. Future basket trials should prospectively enrich thoracic and head and neck cohorts to better define the efficacy and safety of TRK inhibition in these populations [15,16].

### 5.3. NTRK Fusions: Repotrectinib

Repotrectinib is a next-generation compact macrocyclic TRK inhibitor engineered to overcome solvent-front and gatekeeper mutations that limit the durability of first-generation agents. Its FDA tumor-agnostic approval in 2024 was based on the TRIDENT-1 program (NCT03093116), a global phase I/II multicohort trial that enrolled patients with ROS1-, NTRK-, and ALK-rearranged cancers. For the NTRK cohorts, two efficacy populations contributed to approval: a tyrosine kinase inhibitor (TKI)-naive group (*n* = 40) and a TKI-pretreated group (*n* = 48), both assessed by blinded independent central review [24,31].

Across the overall NTRK cohorts, blinded independent central review objective response rates (ORRs) were 58% in TKI-naive disease (23 of 40) and 50% in TKI-pretreated disease (24 of 48). Thoracic representation was primarily from non-small-cell lung cancer (NSCLC). Within the NSCLC subsets, ORRs were 62% in TKI-naive disease (13 of 21; 95% CI 38–82) and 43% after prior TRK inhibitor exposure (6 of 14; 95% CI 18–71). Twelve-month duration-of-response and progression-free-survival rates in the TKI-naive group were 92% (95% CI 76–100) and 64% (95% CI 43–86), respectively, and in the pretreated group they were 44% (95% CI 1–88) and 23% (95% CI 0–49), respectively [24]. These findings confirm durable disease control in NSCLC, particularly in TKI-naive settings, with meaningful activity after prior TRK inhibition.

The Supplementary Appendix of this trial lists thyroid (*n* = 11) and salivary (*n* = 16) tumors within the broader safety population, but these were not linked to the NTRK efficacy cohorts, so efficacy cannot be attributed to thyroid carcinoma, secretory salivary carcinoma, or sinonasal cancers. No small-cell lung, mesothelioma, thymic, or sinonasal tumors were included in the NTRK efficacy datasets. NTRK-specific intracranial outcomes were not separately reported, although the program demonstrated central nervous system penetration. Safety was acceptable, with common adverse events including dizziness and dysgeusia, and treatment-related discontinuations in 3% of patients [31].

Repotrectinib exemplifies the progress of next-generation TRK inhibition while highlighting persistent evidence gaps in thoracic and head and neck cancers. Its efficacy in NSCLC underscores the value of targeting resistance mutations, but the absence of robust data for thyroid, salivary, or sinonasal primaries mirrors the underrepresentation seen with larotrectinib and entrectinib. Future basket trials should prospectively enrich thoracic and head and neck cohorts to validate tumor-agnostic application in these populations [24,31].

### 5.4. MSI-H/dMMR: Pembrolizumab

Pembrolizumab became the first drug to receive a tumor-agnostic FDA approval on 23 May 2017, for adult and pediatric patients with unresectable or metastatic microsatellite instability-high (MSI-H) or deficient mismatch repair (dMMR) solid tumors that had progressed after prior therapy and lacked satisfactory treatment options [10].

This approval was supported by pooled data from five clinical trials: KEYNOTE-016, KEYNOTE-164, KEYNOTE-158, KEYNOTE-012, and KEYNOTE-028. Across these studies, 149 patients with MSI-H or dMMR tumors were enrolled across 15 cancer types, including 90 with colorectal cancer and 59 with non-colorectal cancers. The overall response rate (ORR) was 39.6% (95% CI, 31.7–47.9) with 7% complete responses. Seventy-eight percent of responses were ongoing at six months or longer, and duration of response ranged from 1.6+ to 22.7+ months. In the non-colorectal cohort of KEYNOTE-158 (*n* = 78), the ORR was 34.3%, median progression-free survival (PFS) was 4.1 months, and median overall survival (OS) was 23.5 months [8].

Thoracic and head and neck representation came primarily from KEYNOTE-158, which included thyroid carcinoma (*n* = 5), salivary carcinoma (*n* = 2), small-cell lung cancer (*n* = 4), mesothelioma (*n* = 4), head and neck squamous cell carcinoma (*n* = 1), and nasopharyngeal carcinoma (NPC) (*n* = 1). No non-small-cell lung or sinonasal cancers were reported. The cohort showed a median duration of response not reached, 12-month PFS 34%, and 12-month OS 62% [8].

Other trials contributed limited thoracic or head and neck evidence. KEYNOTE-016 reported an ORR of 71% (5 of 7) in MSI-H non-colorectal tumors without histology detail [7]. KEYNOTE-164 was restricted to colorectal cancer [9]. KEYNOTE-012 enrolled 60 PD-L1-positive head and neck squamous cell carcinoma patients with an ORR of 18%, but MSI status was not prospectively tested [11]. KEYNOTE-028 enrolled 24 PD-L1-positive endometrial cancer patients, including 1 MSI-H case, with MSI status assessed retrospectively [12].

Pembrolizumab’s tumor-agnostic approval was supported by durable responses across MSI-H and dMMR tumors, but representation of thoracic and head and neck cancers was minimal. Only small numbers of thyroid, salivary, small-cell lung, mesothelioma, head and neck squamous cell carcinoma, and nasopharyngeal carcinoma were included, while non-small-cell lung, thymic, and sinonasal tumors were absent. These findings demonstrate pembrolizumab’s broad efficacy in MSI-H and dMMR disease but highlight the need for histology-enriched studies to define outcomes more reliably in thoracic and head and neck malignancies [7,8,9,10,11,12].

### 5.5. dMMR: Dostarlimab

Dostarlimab-gxly received tumor-agnostic FDA accelerated approval on 17 August 2021, for adults with recurrent or advanced deficient mismatch repair (dMMR) solid tumors that had progressed after prior therapy and had no satisfactory alternative options. The approval was based on the GARNET trial (NCT02715284), a multicohort, single-arm, open-label study. Only the dMMR population formed the regulatory basis for approval [18].

In the pooled dMMR population, the overall response rate (ORR) was 41.6%, with 9% complete responses. Responses were durable, and median duration of response (DoR) was not reached at the time of analysis. The primary dataset, Cohort A1, included 129 patients with dMMR endometrial cancer. At a median follow-up of 16.5 months (interquartile range [IQR] 9.9–24.9), the ORR was 43.5% (95% CI 34–53), with 11 complete responses (10.2%) and 36 partial responses (34.6%). Median DoR was not reached, with 89% of responses ongoing at data cutoff. The disease control rate (DCR) was 63.7% [18].

Cohort F included non-endometrial dMMR tumors, but published analyses did not report outcomes by histology, so inclusion of thoracic or head-and-neck cancers cannot be confirmed. Cohort E, which enrolled patients with non-small-cell lung cancer (NSCLC), was MMR-unselected and therefore not part of the biomarker-defined dataset (GARNET Protocol 4010-01-001).

The safety profile was consistent with other PD-1 inhibitors. In the pooled safety population (*n* = 515), the most frequent treatment-emergent adverse events were anemia (25.6%), nausea (25.0%), and diarrhea (22.5%). Grade 3 or higher treatment-related events occurred in 16.6%, and 5.5% of patients discontinued therapy due to toxicity [18].

Dostarlimab’s tumor-agnostic approval was supported by durable activity in dMMR endometrial cancer and pooled efficacy across dMMR tumors. Unlike pembrolizumab, where thoracic and head-and-neck subgroups were documented, GARNET did not report per-histology denominators. The efficacy of dostarlimab in these cancers therefore remains undefined, underscoring the need for future histology-enriched cohorts that include dMMR thoracic and head-and-neck tumors to establish reliable disease-specific outcomes [18].

### 5.6. TMB-High: Pembrolizumab

Pembrolizumab received tumor-agnostic FDA approval in June 2020 for patients with unresectable or metastatic solid tumors showing a tumor mutational burden (TMB) ≥ 10 mutations per megabase (mut/Mb), as determined by the FoundationOne CDx assay (17). The approval was based on a prespecified retrospective biomarker analysis of the phase II KEYNOTE-158 trial (NCT02628067), which enrolled 1073 patients across ten tumor-specific cohorts. Of 1066 treated patients, 805 (76%) were evaluable for TMB, and 790 (74%) were included in the efficacy population. 102 (13%) were TMB-High (≥10 mut/Mb) and 688 (87%) were non-TMB-High (<10 mut/Mb) [8].

In the TMB-High group, 30 of 102 patients responded, yielding an overall response rate (ORR) of 29% (95% CI, 21–39) with 4% complete and 25% partial responses. Median duration of response (DoR) was not reached (range 2.2+ to 34.8+ months), and 67% of responses were ongoing at one and two years. Median progression-free survival (PFS) was 2.1 months (95% CI, 2.1–4.1) and median overall survival (OS) was 11.7 months (95% CI, 9.1–19.1). The non-TMB-High cohort showed an ORR of 6% (43 of 688) with median DoR 33.1 months, PFS 2.1 months, and OS 12.8 months [8].

Thoracic and head-and-neck representation was minimal. The TMB-High efficacy population included small-cell lung cancer (SCLC, *n* = 34), mesothelioma (*n* = 1), thyroid carcinoma (*n* = 2), and salivary gland carcinoma (*n* = 3). No non-small-cell lung, head-and-neck squamous, sinonasal, or thymic cancers were enrolled. Among SCLC patients, 10 of 34 (29%) responded; salivary gland carcinoma had 1 of 3 responders (33%); both thyroid cases responded (2 of 2, 100%); the single mesothelioma patient did not. Given the small numbers, these results are descriptive only, and no CNS-specific data were reported.

Pembrolizumab’s TMB-High approval validated TMB as a predictive biomarker across tumor types but provided limited evidence for thoracic or head-and-neck cancers. In KEYNOTE-158, only a small number of thyroid and salivary tumors demonstrated responses, and there was no representation from NSCLC, thymic, or sinonasal malignancies. Although exploratory analyses suggested occasional responses in SCLC, these data did not form the basis of the TMB-High approval. The ≥10 mut/Mb threshold may not uniformly predict benefit across histologies due to assay variability and differences in underlying mutational biology. While the approval expanded access to immunotherapy, its evidentiary support in thoracic and head-and-neck cancers remains limited, underscoring the need for histology-specific validation studies [8,17].

### 5.7. BRAF V600E: Dabrafenib + Trametinib 

Dabrafenib plus trametinib received tumor-agnostic FDA approval in 2022 for patients aged 6 years and older with unresectable or metastatic solid tumors harboring a BRAF V600E mutation that had progressed after prior therapy and lacked satisfactory alternatives [23]. The approval was supported by data from the Rare Oncology Agnostic Research (ROAR) basket trial (NCT02034110) and the National Cancer Institute Molecular Analysis for Therapy Choice (NCI-MATCH) Subprotocol H (EAY131-H), with additional evidence from pediatric glioma studies.

In the ROAR trial, the anaplastic thyroid carcinoma (ATC) cohort included 36 patients with BRAF V600E mutations. The investigator-assessed overall response rate (ORR) was 56% (20 of 36; 95% CI 38.1–72.1), including 3 complete responses (8%) and 17 partial responses (47%). Median duration of response (DoR) was 14.4 months (95% CI 7.4—not reached), median progression-free survival (PFS) was 6.7 months (95% CI 4.7–13.8), and median overall survival (OS) was 14.5 months (95% CI 6.8–23.2) [21]. The ATC cohort provided the primary thoracic evidence supporting approval.

The NCI-MATCH Subprotocol H enrolled 35 patients with BRAF V600E-mutated tumors across rare histologies, with 29 included in the efficacy population. The confirmed ORR was 38% (11 of 29; 90% CI 22.9–54.9), median PFS 11.4 months (90% CI 8.4–16.3), and median OS 28.6 months. Among these, 5 patients had lung adenocarcinoma with partial responses, and 1 patient had mandibular ameloblastoma that achieved disease control [22].

Non-small-cell lung, mesothelioma, thymic, salivary, sinonasal, or other head-and-neck primaries were included in registrational datasets.

Dabrafenib plus trametinib’s tumor-agnostic approval was driven by strong efficacy in anaplastic thyroid carcinoma, with an ORR of 56% and median OS 14.5 months, and was supported by NCI-MATCH responses in lung adenocarcinoma and a rare head-and-neck tumor, ameloblastoma. Thoracic and head-and-neck representation remained limited, underscoring the need for histology-enriched studies to define efficacy in these underrepresented malignancies [21,22,23].

### 5.8. RET Fusions: Selpercatinib

Selpercatinib received accelerated FDA approval in September 2022 for patients with advanced RET fusion-positive solid tumors that had progressed after prior therapy and lacked satisfactory alternatives [20]. The approval was supported by the LIBRETTO-001 trial (NCT03157128), a global phase 1/2 basket study.

Between December 2017 and August 2021, 45 patients with RET fusion-positive non-lung and non-thyroid tumors were enrolled, and 41 were evaluable for efficacy [19]. The overall response rate (ORR) was 43.9% (18 of 41; 95% CI, 28.5–60.3), including 2 complete responses (5%) and 16 partial responses (39%). Stable disease occurred in 14 patients (34%), progressive disease in 3 (7%), and 6 (15%) were not evaluable. The median duration of response (DoR) was 24.5 months (95% CI, 9.2–not estimable), and median progression-free survival (PFS) was 13.2 months (95% CI, 7.4–26.2). The 1-year and 2-year PFS rates were 53.1% and 32.1%, respectively, and median overall survival (OS) was 18.0 months (95% CI, 10.7—not estimable), with 1-year OS 66.8% and 2-year OS 47.4% [19].

Salivary gland carcinoma accounted for 4 patients (9%), with an ORR of 50% (2 of 4; 95% CI, 6.8–93.2). Both responders remained on treatment at data cutoff, one exceeding 9 months of DoR. A single pulmonary carcinosarcoma (*n* = 1; 2%) did not respond. No sinonasal, thymic, mesothelioma, non-small-cell lung cancer (NSCLC), or head-and-neck squamous cell carcinoma cases were included, as NSCLC and thyroid carcinoma were studied in separate anchor cohorts.

The safety profile was consistent with prior reports in lung and thyroid cancers. Among 45 patients, the most common grade ≥3 treatment-emergent adverse events were hypertension (22%), elevated alanine aminotransferase (16%), and elevated aspartate aminotransferase (13%). Serious adverse events occurred in 18 patients (40%), and no treatment-related deaths were reported [19].

Selpercatinib’s tumor-agnostic approval demonstrated durable efficacy across RET fusion-positive tumors, but thoracic and head-and-neck data were minimal, confined to four salivary gland carcinomas and one pulmonary carcinosarcoma. With only five patients representing these malignancies, precision of efficacy estimates remains limited. Further histology-enriched validation is needed to confirm the reliability of selpercatinib in thoracic and head-and-neck cancers [19,20].

### 5.9. HER2 Overexpression (IHC 3+): Fam-Trastuzumab Deruxtecan (T-DxD)

Fam-trastuzumab deruxtecan (T-DxD) received tumor-agnostic FDA approval in 2024 for patients with unresectable or metastatic solid tumors exhibiting HER2 IHC 3+ overexpression that had progressed on prior therapy [25]. The approval was based on the DESTINY-PanTumor02 trial, which enrolled 267 patients with HER2-expressing tumors. Across all tumor types, the overall response rate (ORR) was 37.1% (95% CI, 30.1–44.5), with median duration of response (DoR) 11.3 months, median progression-free survival (PFS) 6.9 months, and median overall survival (OS) 13.4 months.

In the other-tumors cohort, which included salivary gland cancers (*n* = 19) and head and neck cancers, the investigator-assessed ORR was 30%, and among centrally confirmed IHC 3+ cases the ORR was 44.4% [25]. No thyroid, sinonasal, or squamous cell head-and-neck cancers were reported.

Thoracic evidence was derived primarily from DESTINY-Lung01, which evaluated HER2-overexpressing non-small-cell lung cancer (NSCLC). In Cohort 1 (6.4 mg/kg; *n* = 49), the ORR was 26.5% (95% CI, 15.0–41.1), median DoR 5.8 months, PFS 5.7 months, and OS 12.4 months. In Cohort 1A (5.4 mg/kg; *n* = 41), results improved modestly with ORR 34.1% (95% CI, 20.1–50.6), including 2 complete responses, median DoR 6.2 months, PFS 6.7 months, and OS 11.2 months [26]. Responses were observed among patients with baseline brain metastases, although intracranial outcomes were not separately reported.

The tumor-agnostic approval of T-DxD was supported by broad efficacy across HER2 IHC 3+ tumors, with thoracic evidence from NSCLC in DESTINY-Lung01 and head-and-neck data limited to salivary gland carcinoma in DESTINY-PanTumor02. While these findings demonstrate meaningful clinical activity, the absence of data in other head-and-neck primaries such as thyroid, sinonasal, and squamous carcinomas highlights the need for histology-enriched studies to validate T-DxD’s role in these populations [25,26,27].

### 5.10. Comparative Outcomes Across Histologies: Summary of Key Takeaways

Across tissue-agnostic approvals, the strength of evidence for thoracic and head and neck (H&N) cancers varies markedly by biomarker. NTRK fusions produced the clearest efficacy signal in secretory salivary carcinoma, where pooled response rates to larotrectinib, entrectinib, and repotrectinib approached 90% [13,14,15,16]. Thyroid carcinoma and NSCLC were represented in small numbers, while SCLC, mesothelioma, thymic, and sinonasal cancers were absent.

For RET fusions, thoracic and H&N data came mainly from LIBRETTO-001, which included 4 salivary gland carcinomas (ORR 50%) and one pulmonary carcinosarcoma without response [19]. NSCLC and thyroid carcinoma were studied separately in anchor cohorts.

BRAF V600E alterations were most clinically relevant in anaplastic thyroid carcinoma, where dabrafenib plus trametinib achieved an ORR 56%, DoR 14.4 months, and OS 14.5 months in ROAR [21]. Additional responses occurred in lung adenocarcinoma and ameloblastoma within NCI-MATCH, but no other thoracic or H&N primaries were represented [22].

In HER2-positive tumors, DESTINY-PanTumor02 included 19 salivary gland cancers (ORR 30%, 44.4% for confirmed IHC 3+) [25]. DESTINY-Lung01 demonstrated activity in NSCLC with ORRs of 26.5% (6.4 mg/kg) and 34.1% (5.4 mg/kg) and median OS 11–12 months [26]. Other H&N subtypes, including thyroid, sinonasal, and squamous carcinomas, were not represented.

MSI-H/dMMR and TMB-High contributed minimally. In pembrolizumab’s MSI-H dataset, only small numbers of thyroid (*n* = 5), salivary (*n* = 2), SCLC (*n* = 4), mesothelioma (*n* = 4), HNSCC (*n* = 1), and nasopharyngeal carcinoma (*n* = 1) were included, with no NSCLC or sinonasal tumors [8,10]. Dostarlimab’s GARNET study did not report histology-specific outcomes [18]. For TMB-High, KEYNOTE-158 excluded NSCLC and HNSCC but included 34 SCLC, 1 mesothelioma, 2 thyroid, and 3 salivary cancers with respective ORRs of 29%, 0%, 100%, and 33% [8,17].

The best-represented thoracic and H&N malignancies across tissue-agnostic approvals include secretory salivary carcinoma (NTRK), salivary duct carcinoma (HER2), NSCLC (RET and HER2), and anaplastic thyroid carcinoma (BRAF). Underrepresented cancers include SCLC, mesothelioma, thymic, sinonasal, and conventional HNSCC, which were absent or sporadic. While RET, NTRK, BRAF, and HER2 show reproducible efficacy in selected subsets, MSI-H and TMB-High provide minimal evidence, and most thoracic and H&N histologies remain without sufficient data to inform practice. Key efficacy outcomes and thoracic/H&N representation across tumor-agnostic approvals are summarized in Table 2, and the detailed histologic distribution across pivotal trials is shown in Table 3.

## 6. Emerging and Investigational Biomarkers in Thoracic and Head & Neck Cancers

This section focuses specifically on emerging biomarkers with plausible or demonstrated relevance to thoracic and head and neck malignancies. To maintain conceptual alignment with tumor-agnostic principles, we distinguish between biomarkers supported by cross-histology oncogenic dependency and agents whose activity remains primarily histology-modulated. Only targets with either documented thoracic or head and neck activity or strong biologic plausibility in these cancers are included. This focused framework ensures relevance to the clinical and translational objectives of this review.

As precision oncology continues to evolve, several emerging biomarkers are showing potential to broaden the reach of tissue-agnostic therapy into thoracic and head and neck cancers. Early cross-histology activity has been observed for FGFR alterations, NRG1 fusions, HER3-directed therapies, POLE/POLD1 mutations, and next-generation TRK and RET inhibitors, with the strongest data so far in NSCLC and selected salivary and sinonasal tumors. Although none have yet achieved FDA approval in a tissue-agnostic context, these targets represent promising avenues for future development. Realizing their clinical potential will depend on histology-enriched basket trials and routine implementation of RNA-based sequencing to capture rare and complex fusions that may otherwise go undetected in thoracic and head and neck malignancies.

### 6.1. KRAS G12C: Sotorasib; Adagrasib; Divarasib; Olomorasib

The advent of KRAS G12C inhibitors marks a breakthrough in targeting one of oncology’s most common and previously “undruggable” mutations. Discovery of a covalently targetable cysteine at codon 12 enabled development of sotorasib, adagrasib, and divarasib, which have achieved meaningful clinical benefit in NSCLC. In CodeBreaK 100, sotorasib produced an objective response rate (ORR) of 37% and progression-free survival (PFS) of 6.8 months, while adagrasib achieved a 43% ORR and demonstrated central nervous system (CNS) activity in KRYSTAL-1 [32]. The next-generation inhibitor divarasib has also shown encouraging early results in NSCLC. Olomorasib, another next-generation KRAS G12C inhibitor, has demonstrated potent and selective covalent binding with early clinical activity and a favorable tolerability profile, further expanding the therapeutic landscape for KRAS G12C-mutant lung cancer. Activity across other tumor types, including colorectal and pancreatic cancers, has been modest, and reports in head and neck malignancies remain limited. These agents confirm the therapeutic value of allele-specific KRAS targeting in lung cancer while emphasizing the need for basket trials and histology-enriched studies to explore their potential across broader thoracic and head and neck disease contexts.

### 6.2. Fibroblast Growth Factor Receptor (FGFR) Alterations: Pemigatinib; Infigratinib; Futibatinib; Bemarituzumab; Alofanib

Fibroblast growth factor receptor (FGFR) alterations represent a promising class of emerging tissue-agnostic targets with growing relevance in thoracic and head and neck (H&N) cancers [33]. Activating fusions, mutations, and amplifications in FGFR drive MAPK and PI3K/AKT signaling and are most frequently seen in sinonasal squamous, salivary gland, and rare NSCLC subsets [34,35]. Further clinical experience has been summarized in recent futibatinib reviews [36]. The phase II RAGNAR trial (NCT04083976) demonstrated an objective response rate (ORR) of 29.5% and durable benefit across more than 20 tumor types, establishing FGFR as a biologically credible cross-histology biomarker. Early futibatinib studies, such as FOENIX-101, confirmed activity across multiple FGFR-altered tumors [37]. Additional FGFR2-selective agents such as alofanib have shown early-phase activity [38]. Although responses in thoracic and H&N malignancies were limited, the findings confirm the feasibility of FGFR-targeted therapy [38]. Broader adoption of RNA-based fusion testing and histology-enriched basket trials focusing on sinonasal and salivary cancers will be key to defining the true therapeutic potential of FGFR inhibition in these populations [1].

### 6.3. Neuregulin-1 (NRG1) Fusions: Zenocutuzumab

Neuregulin-1 (NRG1) fusions are rare but therapeutically actionable oncogenic drivers that activate HER3 signaling through HER2–HER3 dimerization [39]. They are most prevalent in invasive mucinous adenocarcinoma of the lung, particularly KRAS wild-type tumors, and have been reported in salivary gland carcinomas, supporting biologic plausibility in head and neck cancers. The bispecific antibody zenocutuzumab (MCLA-128) provided the first prospective evidence of efficacy in the eNRGy trial (NCT02912949), achieving an overall response rate (ORR) of 34%, median duration of response 12.9 months, and progression-free survival (PFS) 5.6 months, including an ORR of 42% in NSCLC [39]. Zenocutuzumab recently received regulatory approval for NRG1 fusion-positive pancreatic cancer, supported by durable responses in this highly lethal disease, further validating the therapeutic relevance of HER3 blockade across distinct epithelial lineages. Responses were observed regardless of fusion partner, confirming HER3 dependency as the therapeutic mechanism. While data for salivary and other head and neck tumors remain limited, zenocutuzumab establishes proof-of-concept for targeting NRG1 fusions. Accurate identification requires RNA-based sequencing, as DNA-only panels frequently miss these rearrangements. Zenocutuzumab thus represents a promising tissue-agnostic therapy with validated efficacy in lung cancer and emerging relevance in salivary primaries.

### 6.4. Human Epidermal Growth Factor Receptor 3 (HER3) Antibody–Drug Conjugates: Patritumab Deruxtecan

HER3 (ERBB3) is an emerging therapeutic target that mediates oncogenic signaling through HER2-driven activation of MAPK and PI3K/AKT pathways and contributes to EGFR inhibitor resistance in NSCLC. The antibody–drug conjugate patritumab deruxtecan (HER3-DXd) links a fully human anti-HER3 antibody to a topoisomerase I payload. In early-phase studies, it produced meaningful responses in heavily pretreated EGFR-mutant NSCLC, and the HERTHENA-Lung01 trial (NCT04619004) confirmed objective response rates (ORR) of 30–40% with durable benefit, though interstitial lung disease (ILD)/pneumonitis was a notable toxicity [40,41]. The confirmatory overall survival (OS) analysis was negative, and the final OS endpoint was not met. Despite these results, the therapy demonstrated consistent tumor shrinkage and prolonged disease control, but the US BLA submission was ultimately withdrawn in May 2025, leaving HER3-DXd investigational. In head and neck cancers, HER3 is expressed in salivary duct carcinoma and subsets of HNSCC, but clinical validation is lacking. HER3-DXd establishes proof-of-concept for HER3 as a cross-histology target in NSCLC and warrants further study in salivary and squamous head and neck malignancies through basket or histology-enriched trials.

### 6.5. RET Fusions: Pralsetinib

Pralsetinib is a highly selective RET inhibitor with potent activity in RET fusion-positive malignancies. The ARROW trial (NCT03037385) demonstrated an objective response rate (ORR) >60% and median duration of response >17 months in non-small-cell lung cancer (NSCLC), leading to FDA approval, along with durable responses in RET-altered thyroid cancers [42,43]. Although ARROW’s basket design included multiple histologies, only a few non-lung and non-thyroid tumors were enrolled, preventing a tissue-agnostic label, though isolated responses in salivary gland carcinoma were reported [43]. Because RET fusions are rare outside NSCLC and thyroid cancer, RNA-based sequencing is preferred for detecting uncommon fusion partners with higher sensitivity than DNA-based methods. Pralsetinib demonstrates strong efficacy in lung and thyroid cancers and emerging potential in rare head and neck malignancies, warranting larger, histology-enriched trials to explore broader tissue-agnostic application.

### 6.6. Next-Generation TRK Inhibitors: Selitrectinib (LOXO-195); Repotrectinib

Next-generation TRK inhibitors were developed to overcome resistance mechanisms that limit first-generation agents like larotrectinib and entrectinib, including solvent-front, gatekeeper, and xDFG mutations that restore signaling. Selitrectinib (LOXO-195) was the first designed to retain potency against these variants and has achieved meaningful responses in pediatric and adult patients with TRK fusion-positive cancers after prior TRK inhibitor therapy [15]. Repotrectinib, a compact macrocyclic inhibitor of TRK, ROS1, and ALK, demonstrates strong central nervous system (CNS) penetration and potent activity against resistance mutations. In the TRIDENT-1 trial (NCT03093116), it produced durable responses in previously treated NTRK fusion-positive tumors, supporting FDA approvals for ROS1-positive NSCLC (2023) and a tissue-agnostic NTRK indication (2024) [24]. Within thoracic and head and neck malignancies, these drugs are most relevant for secretory salivary carcinoma, where NTRK fusions are pathognomonic, and for NTRK-positive NSCLC. Together, selitrectinib and repotrectinib exemplify the next phase of TRK inhibition, maintaining tissue-agnostic efficacy in the resistance setting.

### 6.7. DNA Polymerase Epsilon and Delta 1 (POLE and POLD1) Mutations: Pembrolizumab; Nivolumab

Expanding the scope of immune biomarkers, POLE and POLD1 mutations represent a rare but mechanistically powerful class associated with ultramutated tumors highly sensitive to PD-1 blockade. Loss of exonuclease proofreading function in these polymerases produces exceptionally high tumor mutational burden (TMB) and abundant neoantigens, driving strong responses to checkpoint inhibitors [44]. Although uncommon, these alterations are documented in NSCLC and head and neck squamous carcinomas, where case-level reports describe deep and durable responses to pembrolizumab and nivolumab [45,46]. Broader data from KEYNOTE-158, CheckMate 227, and TMB-high analyses support this biology, confirming PD-1 benefit in ultramutated tumors [8,47,48]. In thoracic and H&N cancers, POLE/POLD1 mutations occur in both adenocarcinoma and squamous subsets, including oral, oropharyngeal, and sinonasal primaries, though evidence remains limited to small cohorts. Ongoing basket and registry trials are expected to clarify cross-histology efficacy. POLE/POLD1 mutations align with the TMB-response paradigm and may ultimately emerge as validated tissue-agnostic biomarkers once confirmed in larger, histology-enriched studies.

### 6.8. TP53 Y220C: Rezatapopt 

Among tumor-suppressor targets, TP53 Y220C represents a unique opportunity for pharmacologic reactivation. This missense mutation creates a surface pocket that destabilizes p53 but also provides a druggable cavity for small-molecule binding. Rezatapopt (PC14586), the first clinical-stage Y220C-selective compound, restores wild-type p53 function by stabilizing the mutant protein. In the PYNNACLE trial (NCT04585750), interim results showed an objective response rate of 34%, with durable responses particularly in ovarian, endometrial, and select thoracic malignancies, including NSCLC [49]. Although evidence in head and neck cancers remains preliminary, the biologic rationale supports activity wherever this mutation occurs. If validated in larger, histology-enriched cohorts, rezatapopt could establish a proof of concept for tumor suppressor reactivation as a novel, tissue-agnostic therapeutic strategy.

### 6.9. ALK/ROS1 Rearrangements: Crizotinib; Lorlatinib; Entrectinib

ALK and ROS1 rearrangements create potent oncogenic drivers across several malignancies, most prominently in non-small-cell lung cancer (NSCLC). Entrectinib (RXDX-101) is a multitargeted inhibitor of ALK, ROS1, and TRK kinases optimized for central nervous system (CNS) penetration; however, its ALK activity is limited, and clinically meaningful ALK inhibition is achieved primarily with established agents such as crizotinib and lorlatinib. In pooled analyses of the ALKA-372-001 and STARTRK-1 trials, ROS1-rearranged tumors (mostly NSCLC) achieved an objective response rate (ORR) of 86% and ALK-rearranged tumors an ORR of 57%, with median duration of response exceeding 17 months and documented intracranial responses [15,16]. The ongoing STARTRK-2 (NCT02568267) basket study continues to demonstrate robust, durable activity across histologies, confirming CNS efficacy. Thoracic relevance is well established in NSCLC, while head and neck tumors with ALK or ROS1 fusions are exceedingly rare, with only isolated case-level evidence. Although entrectinib provides meaningful ROS1 and TRK inhibition with good CNS penetration, ALK-directed therapy remains best defined by crizotinib, lorlatinib, and other next-generation ALK TKIs, with entrectinib contributing limited supplemental activity in this space.

### 6.10. cMET Inhibitors and MET-Directed Therapies: Capmatinib; Tepotinib; Savolitinib; Telisotuzumab Vedotin

MET pathway dysregulation, through exon 14 skipping mutations, gene amplifications, or protein overexpression, promotes oncogenesis via MAPK and PI3K/AKT activation. These alterations occur in 3–4% of NSCLC adenocarcinomas and in smaller subsets of sarcomatoid and squamous carcinomas, with rare occurrences in head and neck (H&N) and salivary tumors.

Capmatinib (INC280) was the first highly selective MET inhibitor approved, based on GEOMETRY mono-1 (NCT02414139), which demonstrated an objective response rate (ORR) of 68% and median duration of response (DoR) of 12.6 months in untreated MET exon 14-skipping NSCLC, along with intracranial responses in nearly half of patients with brain metastases [50]. Tepotinib, evaluated in the VISION trial (NCT02864992), showed an ORR of 46% and DoR of 11.1 months with consistent efficacy across tissue and liquid biopsy cohorts and meaningful CNS activity [51]. Savolitinib demonstrated similar activity (ORR 49%) in MET exon 14-altered NSCLC and is being investigated in combination with osimertinib to overcome MET-mediated resistance in EGFR-mutant disease [51].

A new therapeutic class has emerged with telisotuzumab vedotin (Teliso-V), a c-MET-directed antibody–drug conjugate recently approved for c-MET-overexpressing, EGFR wild-type NSCLC following platinum chemotherapy and immunotherapy. Teliso-V delivers a microtubule-disrupting payload via a MET-targeted antibody and has demonstrated meaningful and durable activity, expanding the scope of MET-targeted therapy beyond canonical exon 14-skipping disease and into protein-overexpressing tumors.

While MET overexpression is documented in subsets of HNSCC and salivary gland carcinomas, prospective data remain minimal. MET TKIs and Teliso-V validate MET as a therapeutically actionable target in NSCLC and highlight the need for basket trials to clarify their role in underrepresented thoracic and head and neck malignancies.

### 6.11. PARP Inhibitors: Olaparib; Niraparib; Talazoparib

Poly (ADP-ribose) polymerase (PARP) inhibitors leverage synthetic lethality in tumors with homologous recombination repair (HRR) deficiencies, particularly those with BRCA1/2, PALB2, RAD51, or ATM mutations. By inhibiting PARP1/2 and trapping PARP–DNA complexes, they induce unrepaired DNA damage and tumor cell death.

Olaparib first demonstrated this principle, improving progression-free survival in germline BRCA-mutated ovarian, pancreatic (POLO), and prostate cancers (PROfound) [52,53]. Niraparib (PRIMA) extended benefit to biomarker-unselected ovarian cancer, and talazoparib (EMBRACA) confirmed efficacy in BRCA-mutant breast cancer [54,55]. Ongoing KEYLYNK-007 evaluates olaparib plus pembrolizumab across tumor types.

In thoracic and head and neck (H&N) cancers, HRR alterations are uncommon, but case reports describe durable responses in BRCA2- or PALB2-mutated NSCLC and salivary tumors. While not yet tumor-agnostic, PARP inhibitors show reproducible activity in HRR-deficient cancers across organs. Future progress will depend on basket trials and combination strategies to confirm their role in rare thoracic and H&N malignancies.

### 6.12. Next-Generation RET Inhibitors: HM06; HS-10365

Acquired resistance remains a major limitation of first-generation TRK and RET inhibitors, often arising from solvent-front and gatekeeper mutations such as RET G810 or NTRK1 G595R, which restore kinase activity. To overcome these mechanisms, next-generation inhibitors have been developed. For TRK, agents such as selitrectinib and repotrectinib have demonstrated clinical efficacy after progression on larotrectinib or entrectinib [56].

For RET, TPX-0046 was an early next-generation macrocyclic RET and SRC inhibitor designed to retain potency against solvent-front and gatekeeper substitutions, but its clinical development has been discontinued after early phase studies. The next-generation RET inhibitors that remain in active development are HM06 and HS-10365. Both agents are engineered to overcome RET G810 solvent-front mutations and other refractory resistance mechanisms while maintaining selectivity and central nervous system penetration [56].

In thoracic and head and neck cancers, RET fusions remain rare but clinically significant, particularly in non-small-cell-lung cancer and thyroid carcinoma, where on-target resistance has been well characterized. Similar resistance biology likely applies to salivary secretory carcinoma, where sequential inhibition with next-generation TRK agents has been effective. As additional data mature, HM06 and HS-10365 may provide the next wave of RET-selective therapy capable of restoring response in patients who progress on selpercatinib or pralsetinib, and may enable longer-term precision treatment across histologies [56]. Additional emerging and investigational tumor-agnostic biomarkers relevant to thoracic and head and neck cancers are summarized in Table 4.

## 7. Methodological Limitations and Evidence Gaps

Interpretation of tumor-agnostic approvals in thoracic and head and neck (H&N) cancers is limited by multiple methodological and diagnostic challenges. The most significant issue is the underrepresentation of these histologies in registrational basket trials, which typically enrolled many tumor types without prespecified thoracic or H&N cohorts. As a result, subgroup analyses were often absent or based on single-digit sample sizes, restricting the reliability of efficacy estimates for outcomes such as durability of response, intracranial control, and long-term safety [13,16,19].

Most pivotal trials were non-randomized and relied primarily on objective response rate (ORR) to secure accelerated approval. While effective for proofs of concept, this design left progression-free and overall survival largely undefined for thoracic and H&N cancers. Inconsistent trial endpoints further complicate cross-study comparisons and limit generalizability [8,10].

Diagnostic variability also contributes to uncertainty. Tumor mutational burden (TMB) remains unstandardized across sequencing platforms, with differences in assay design and bioinformatics pipelines producing inconsistent classifications in NSCLC and HNSCC. Similarly, fusion detection depends heavily on the testing method; DNA-based next-generation sequencing (NGS) can miss complex RET, NTRK, or NRG1 fusions more reliably detected with RNA-based sequencing, leading to underestimation of prevalence and missed treatment opportunities [19].

Another limitation is the inconsistent evaluation of intracranial efficacy, despite the high incidence of brain metastases in NSCLC. Only entrectinib and selpercatinib consistently reported CNS outcomes, while most other tumor-agnostic studies excluded patients with active brain disease or lacked biomarker-specific CNS data [16,20].

Entire histologies, including thymic carcinoma, mesothelioma, small-cell lung cancer, sinonasal carcinoma, and most HNSCC subtypes, were absent or minimally represented, leaving no prospective data to guide therapy [7,28]. Clinicians must extrapolate from pooled pan-cancer datasets that lack histology-specific validation.

Future trial design should address these limitations by embedding histology-enriched thoracic and H&N cohorts within basket and umbrella studies, prespecifying CNS endpoints, and adopting centralized RNA-based sequencing to harmonize fusion detection and enhance comparability. Without these refinements, clinical decisions in thoracic and H&N oncology will continue to rely on extrapolated, heterogeneous evidence rather than robust, disease-specific data.

## 8. Real-World Application and Challenges in Thoracic and Head & Neck Oncology

Applying tumor-agnostic therapies in thoracic and head and neck (H&N) cancers requires careful clinical judgment, as evidence from registrational trials is uneven and real-world survival data remain limited. The clearest areas of benefit are those supported by robust prospective evidence and measurable activity in these histologies. Selective RET inhibitors such as selpercatinib are now standards of care for RET-rearranged non-small-cell lung cancer (NSCLC) and thyroid carcinoma, supported by LIBRETTO-001, which confirmed durable responses and intracranial activity [19,20]. TRK inhibitors including larotrectinib and entrectinib have shown near-universal efficacy in secretory salivary carcinoma, with pooled response rates approaching 90% [13,16]. Dual BRAF and MEK inhibition with dabrafenib plus trametinib has also redefined outcomes in BRAF V600E-mutated anaplastic thyroid carcinoma, achieving a 56% response rate and a median overall survival of 14.5 months in the ROAR trial [21]. In these well-characterized settings, tumor-agnostic therapies can be applied with confidence and have become part of standard treatment algorithms. In settings where thoracic and head and neck representation is sparse or absent in registrational trials, clinical decision-making should be guided by three principles. First, confirm high-confidence biomarker dependency using validated assays, ideally incorporating RNA-based fusion testing when relevant. Second, assess whether the biomarker has demonstrated lineage-independent oncogenic addiction in other tumor types rather than relying solely on pooled response rates. Third, prioritize enrollment in clinical trials or registry studies whenever feasible to contribute disease-specific evidence. When these options are unavailable, treatment decisions should be individualized through multidisciplinary discussion, balancing biologic plausibility, available alternatives, performance status, and toxicity profile.

Beyond these indications, the evidence base remains limited. MSI-H and TMB-High subsets occur in fewer than 1% of NSCLC and HNSCC, and corresponding approvals were derived from pooled pan-cancer analyses with minimal representation of these histologies [7,8,10,18]. HER2-directed therapy with fam-trastuzumab deruxtecan has shown efficacy in NSCLC and salivary duct carcinoma, but other head and neck primaries including thyroid, sinonasal, and squamous cell carcinomas were not represented in pivotal studies [25,26,27]. Small-cell lung cancer, thymic carcinoma, and mesothelioma also contributed little or no data, leaving clinicians without disease-specific evidence to guide therapy in these populations. In these cases, extrapolation from pan-cancer results is often necessary, although participation in clinical trials should remain the preferred approach whenever possible.

Real-world barriers further constrain implementation. Access to comprehensive next-generation sequencing (NGS) is inconsistent, and RNA-based assays that are required for accurate fusion detection are often unavailable outside major academic centers [28]. Cost, insurance approval delays, and long turnaround times create additional disparities in testing and treatment access. Toxicities also pose practical challenges in community settings. Interstitial lung disease with HER2-directed antibody–drug conjugates requires close monitoring, while metabolic toxicities such as hyperphosphatemia remain frequent with investigational FGFR inhibitors [25,26,27].

National health insurance structures and reimbursement policies further influence implementation. Coverage for comprehensive next-generation sequencing panels varies across public and private payers, and RNA-based fusion testing is not uniformly reimbursed despite its diagnostic importance. In many healthcare systems, access to broad molecular profiling remains limited to tertiary centers, restricting equitable identification of rare but actionable alterations. Drug affordability presents an additional barrier, particularly for antibody–drug conjugates and next-generation targeted therapies, where out-of-pocket costs may delay or prevent initiation. These systemic factors significantly shape real-world uptake of tumor-agnostic therapies and must be considered when interpreting trial-level efficacy in routine practice.

In summary, the real-world application of tumor-agnostic therapies in thoracic and head and neck cancers is strongest for RET-rearranged NSCLC and thyroid carcinoma, NTRK fusion-positive salivary carcinoma, and BRAF V600E-mutated anaplastic thyroid carcinoma, where prospective data support routine use. Outside these settings, patient numbers remain small, histology-specific outcomes are undefined, and access barriers persist. Until registries and histology-enriched basket studies expand the evidence base, clinicians should apply tumor-agnostic therapies selectively, prioritize enrollment in clinical trials, and advocate for equitable access to advanced molecular testing to ensure that all eligible patients can benefit from precision oncology.

## 9. Conclusions and Future Directions in Thoracic and Head & Neck Oncology

Thoracic and head and neck (H&N) malignancies remain among the most difficult cancers to treat, characterized by advanced-stage presentation and historically poor survival despite multimodal therapy. The integration of next-generation sequencing (NGS) into clinical practice has enabled systematic identification of oncogenic drivers and laid the foundation for tissue-agnostic drug development. Clinically mature advances include selpercatinib for RET fusions, TRK inhibitors for NTRK fusions (notably secretory salivary carcinoma), and dabrafenib plus trametinib for BRAF V600E (particularly anaplastic thyroid carcinoma). In select subsets, pembrolizumab for TMB-High tumors and fam-trastuzumab deruxtecan for HER2 IHC 3+ tumors have further expanded precision options. In these contexts, biomarker-directed therapy has transformed outcomes and provided meaningful survival benefits for diseases that once had few effective treatments.

For other approvals, the impact on thoracic and H&N cancers remains limited by sparse representation in pivotal datasets. MSI-H and TMB-High subsets occur in fewer than 1% of NSCLC, HNSCC, and related cancers, while sinonasal, thymic, and small-cell histologies were absent or underrepresented. This lack of data forces clinicians to rely on extrapolation from pan-cancer cohorts without histology-specific validation, creating uncertainty regarding efficacy, durability, and safety in these populations.

To optimize patient selection and therapeutic benefit, clinical practice should adopt a testing-first approach. All patients with advanced thoracic or head-and-neck malignancies should undergo comprehensive DNA- and RNA-based NGS to identify oncogenic drivers and fusion events. In lung adenocarcinoma specifically, c-MET protein overexpression should be assessed using immunohistochemistry, with ≥50% of tumor cells demonstrating 3+ staining as the clinically meaningful threshold. HER2 testing should similarly rely on immunohistochemistry with a clear IHC 3+ requirement for eligibility. TMB should be measured using FDA-approved assays such as FoundationOne CDx, interpreted cautiously given cross-tumor variability. In addition to these biomarkers, TROP2 expression represents an emerging target that may guide antibody–drug conjugate selection in both thoracic and head-and-neck cancers. Whenever feasible, patients should be referred to basket or umbrella trials that prespecify thoracic and head-and-neck cohorts to improve representation of these historically under-studied populations.

Future research must emphasize histology-enriched trial designs within pan-tumor programs to generate reliable, disease-specific estimates of benefit. Diagnostic standardization is equally important, including harmonized TMB thresholds and wider adoption of RNA-based fusion testing to ensure accurate case detection. Real-world registries are needed to capture safety signals such as interstitial lung disease with antibody–drug conjugates, to track resistance mechanisms, and to define the durability of benefit in rare subgroups. Emerging tools such as liquid biopsy and circulating tumor DNA (ctDNA) monitoring hold promise for refining patient selection, detecting molecular relapse, and guiding therapeutic sequencing. In unresectable stage III NSCLC, ctDNA-based minimal residual disease (MRD) detection during chemoradiotherapy and durvalumab consolidation has shown strong prognostic value, with detectable ctDNA in the first four months post-CRT associated with significantly shorter progression-free survival [57].

These insights highlight both the progress achieved and the challenges that persist. Comprehensive molecular profiling should be standard for all patients with thoracic and H&N cancers to ensure equitable access to tumor-agnostic therapies where benefit is established. At the same time, greater clinical trial participation, histology-enriched study design, and systematic real-world validation are essential to strengthen the evidence base. Only through these efforts can tumor-agnostic therapies be reliably integrated into durable, evidence-based standards of care for thoracic and head and neck oncology.

## Figures and Tables

**Table 1:** Timeline of FDA Tissue-Agnostic Approvals (2017–2024) Across Nine Therapies and Six Biomarker Classes.

Year	Drug(s)	Biomarker	Key Trial(s)	FDA Approval Note
2017	Pembrolizumab	MSI-H/dMMR	KEYNOTE-016, 164, 158	First tumor-agnostic FDA approval (immune checkpoint inhibitor)
2018	Larotrectinib	NTRK fusion	LOXO-TRK-14001, SCOUT, NAVIGATE (pooled phase I/II)	First selective TRK inhibitor approval
2019	Entrectinib	NTRK fusion	STARTRK-1, 2; ALKA-372-001	Expanded TRK inhibitor option; demonstrated CNS activity
2020	Pembrolizumab	TMB-H (≥10 mut/Mb)	KEYNOTE-158	First biomarker-based approval using TMB as a pan-cancer predictor
2021	Dostarlimab	dMMR	GARNET	Second immune checkpoint inhibitor approval for MSI-H/dMMR
2022	Dabrafenib + Trametinib	BRAF V600E	ROAR basket trial; NCI-MATCH Subprotocol H	First tumor-agnostic BRAF/MEK inhibitor approval
2022	Selpercatinib	RET fusion	LIBRETTO-001	First tumor-agnostic RET inhibitor approval
2024	Repotrectinib	NTRK fusion	TRIDENT-1 (phase I/II)	First next-generation TRK inhibitor with tumor-agnostic approval
2024	Fam-trastuzumab deruxtecan (T-DxD)	HER2-positive (IHC 3+) tumors	DESTINY-PanTumor02; DESTINY-Lung01; DESTINY-CRC02	First HER2-directed ADC with tumor-agnostic approval

Abbreviations: MSI-H = microsatellite instability-high; dMMR = deficient mismatch repair; TMB-H = tumor mutational burden-high; NTRK = neurotrophic tropomyosin receptor kinase; RET = rearranged during transfection; BRAF = B-Raf proto-oncogene, serine/threonine kinase; MEK = mitogen-activated protein kinase kinase; HER2 = human epidermal growth factor receptor 2; IHC = immunohistochemistry; ADC = antibody–drug conjugate; FDA = U.S. Food and Drug Administration.

**Table 2:** Tumor-Agnostic Approvals: Efficacy and Thoracic/H&N Representation.

Drug (Approval Year)	Dataset Referenced in Manuscript	Total N (Dataset)	ORR (%)	Median DoR	Median PFS	Median OS	Thoracic Representation	H&N Representation
Pembrolizumab (2017)	Pooled KEYNOTE approval dataset	149	39.6	1.6+–22.7+ mo (78% ≥6 mo)	4.1 mo	23.5 mo	SCLC (*n* = 4); Mesothelioma (*n* = 4)	Thyroid (*n* = 5); Salivary (*n* = 2); HNSCC (*n* = 1); NPC (*n* = 1)
Pembrolizumab (2020)	KEYNOTE-158 TMB-High subgroup	102	29	Not reached	2.1 mo	11.7 mo	SCLC (*n* = 34); Mesothelioma (*n* = 1)	Thyroid (*n* = 2); Salivary (*n* = 3)
Larotrectinib (2018)	Integrated LOXO-TRK/SCOUT/NAVIGATE	55	75	Not reached	Not reached	Not reported	Lung tumors (*n* = 4)	Salivary (*n* = 12); Thyroid (*n* = 5)
Entrectinib (2019)	STARTRK integrated dataset	54	57	10.4 mo	11.2 mo	21.0 mo	NSCLC (*n* = 10)	Salivary (*n* = 7); Thyroid (*n* = 5)
Repotrectinib (2024)	TRIDENT-1 NTRK cohorts	40 naïve; 48 pretreated	58 (naïve); 50 (pretreated)	NE (naïve); 9.9 mo (pretreated)	30.3 mo (naïve); 7.4 mo (pretreated)	Not reported	NSCLC subset: 62% ORR naïve; 43% pretreated	Thyroid (*n* = 11); Salivary (*n* = 16)
Dostarlimab (2021)	GARNET dMMR pooled	209	41.6	34.7 mo	6.9 mo	Not reached	Not reported by histology	Not reported by histology
Dabrafenib + Trametinib (2022)	ROAR ATC cohort	36 (ATC)	56	14.4 mo	6.7 mo	14.5 mo	Lung adenocarcinoma in MATCH (*n* = 5)	ATC (*n* = 36)
Selpercatinib (2022)	LIBRETTO-001 tumor-agnostic cohort	41	43.9	24.5 mo	13.2 mo	18.0 mo	Pulmonary carcinosarcoma (*n* = 1)	Salivary (*n* = 4)
Fam-trastuzumab deruxtecan (2024)	DESTINY-PanTumor02	267	37.1	11.3 mo	6.9 mo	13.4 mo	NSCLC cohorts in Lung01	Salivary (*n* = 19)

**Table 3:** Tumor-Agnostic Approvals: Thoracic and Head & Neck Representation Across Pivotal Trials.

Drug (Approval Year)	Pivotal Trials	Total N (Efficacy Set)	NSCLC	SCLC	Mesothelioma	Thymic	Thyroid (PTC/ATC/MTC)	Salivary (MASC/Ductal)	Sinonasal	HNSCC	CNS Outcomes (Biomarker-Specific)
Larotrectinib (2018)	LOXO-TRK-14001 (NCT02122913); SCOUT (NCT02637687); NAVIGATE (NCT02576431)	55	4	0	0	0	5	12	0	0	Baseline CNS 1/55 (2%); per-histology efficacy NR
Entrectinib (2019)	ALKA-372-001; STARTRK-1; STARTRK-2 (integrated)	54	10	0	0	0	5	7	0	0	Intracranial ORR 55% in pts with baseline brain mets (*n* = 12); median iPFS 14 mo
Repotrectinib (2023/24)	TRIDENT-1 (NCT03093116)	88 (40 naïve, 48 pretreated)	≈21/40 naïve; ≈14/48 pretreated	0	0	0	0	0	0	0	NTRK-cohort intracranial outcomes NR
Pembrolizumab (2017)	KEYNOTE-016; -164; -158; -012; -028 (pooled)	149	0	4	4	0	5	2	0	1	Subgroup CNS outcomes NR; overall non-CRC outcomes reported only
Dostarlimab (2021)	GARNET A1 (dMMR endometrial cancer [EC]), A2 (MMRp EC), F (non-EC dMMR/MSI-H), E (NSCLC unselected)	A1: 141; A2: 161; F: NR	NR (E unselected; excluded)	NR	NR	NR	0	0	0	0	A1 overall outcomes reported; F per-histology outcomes NR
Pembrolizumab (2020)	KEYNOTE-158 biomarker analysis; FoundationOne CDx	102/790 (13%)	0 (excluded)	34	1	0	2	3	0	0	CNS outcomes NR
Dabrafenib + Trametinib (2022)	ROAR (NCT02034110); NCI-MATCH (EAY131-H); pediatric glioma	ROAR: 206; MATCH: 29 eval	5 (MATCH, lung adeno)	0	0	0	36 (ATC, ROAR)	0	0	0	CNS outcomes NR for solid tumor cohorts
Selpercatinib (2022)	LIBRETTO-001 (NCT03157128) tumor-agnostic cohort (non-lung/thyroid)	41/45	1 (pulmonary carcinosarcoma)	0	0	0	Excluded (thyroid in anchors)	4	0	0	Subgroup CNS outcomes NR
Fam-trastuzumab deruxtecan, T-DxD (2024)	DESTINY-PanTumor02 (NCT04482309); DESTINY-Lung01 (NCT03505710); DESTINY-CRC02 (NCT04744831)	PanTumor02: 267; Lung01: 90; CRC02: 122	49 (6.4 mg/kg)/41 (5.4 mg/kg)	0	0	0	NR	19 salivary cancers; ORR 30% overall, 44.4% in IHC 3+	0	0	Subgroup CNS outcomes NR

**Table 4:** Emerging and Investigational Tumor-Agnostic Biomarkers in Thoracic and Head & Neck Cancers.

Biomarker	Thoracic/H&N Relevance	Lead Investigational Agents	Key Trial(s)/Evidence	Notes & Challenges
Fibroblast Growth Factor Receptor (FGFR) Alterations	Sinonasal carcinoma (FGFR3–TACC3 fusions); rare NSCLC and salivary carcinomas	Pemigatinib; Infigratinib; Futibatinib; Bemarituzumab; Alofanib	FIGHT-101; futibatinib basket cohorts	Proof of concept from cholangiocarcinoma and urothelial carcinoma; thoracic/H&N data sparse; RNA-based NGS preferred for fusion detection
Neuregulin-1 (NRG1) Fusions	NSCLC invasive mucinous adenocarcinoma (pathognomonic); rare salivary carcinomas	Zenocutuzumab (HER2/HER3 bispecific antibody)	eNRGy1 trial	Extremely rare (<1%); requires RNA-based sequencing; early cross-histology efficacy signals, but thoracic/H&N cohorts small
RET Fusions (Histology-Specific)	NSCLC (1–2% adenocarcinoma); thyroid carcinomas; rare salivary and sinonasal tumors	Pralsetinib	ARROW	FDA approvals histology-restricted (NSCLC, thyroid); pan-cancer potential limited by small non-lung/thyroid cohorts
Next-Generation RET Inhibitors	RET-driven NSCLC; thyroid carcinoma (papillary, anaplastic, medullary)	TPX-0046	Early-phase resistance trials	Designed to overcome solvent-front/gatekeeper mutations; potential to extend durability of RET inhibition
Next-Generation TRK Inhibitors	Secretory salivary carcinoma (pathognomonic); rare NSCLC adenocarcinoma	Selitrectinib (LOXO-195); Repotrectinib	Phase I/II studies; Repotrectinib FDA-approved (2023/24)	Addresses resistance to larotrectinib/entrectinib; Repotrectinib first next-gen TRK with tumor-agnostic approval; strong CNS penetration
HER3 Antibody–Drug Conjugates (ADCs)	NSCLC (EGFR-mutant post-TKI); exploratory in salivary duct carcinoma and HNSCC	Patritumab deruxtecan	HERTHENA-Lung01	~40% ORR in EGFR-mutant NSCLC; ILD/pneumonitis risk; role in H&N malignancies remains investigational
DNA Polymerase Epsilon/Delta 1 (POLE/POLD1) Mutations	Rare NSCLC adenocarcinoma/squamous; ultramutated HNSCC (oral cavity, oropharynx, larynx, sinonasal)	Immune checkpoint inhibitors (Pembrolizumab; Nivolumab; others)	Case reports; small series	Strong ICI sensitivity; compelling biology; very rare prevalence; prospective basket validation needed

## Data Availability

No new data were created or analyzed in this study. Data sharing is not applicable to this article.

## Abbreviations

The following abbreviations are used in this manuscript:

ADC	Antibody–Drug Conjugate
ALK	Anaplastic Lymphoma Kinase
ATC	Anaplastic Thyroid Carcinoma
BRAF	B-Raf Proto-Oncogene, Serine/Threonine Kinase
CDx	Companion Diagnostic
CI	Confidence Interval
CNS	Central Nervous System
CRC	Colorectal Cancer
CR	Complete Response
ctDNA	Circulating Tumor DNA
DoR	Duration of Response
EGFR	Epidermal Growth Factor Receptor
FDA	U.S. Food and Drug Administration
FGFR	Fibroblast Growth Factor Receptor
GTP/GDP	Guanosine Triphosphate/Guanosine Diphosphate
HER2	Human Epidermal Growth Factor Receptor 2
HER3	Human Epidermal Growth Factor Receptor 3
HNSCC	Head and Neck Squamous Cell Carcinoma
HRR	Homologous Recombination Repair
H&N	Head and Neck
IHC	Immunohistochemistry
ILD	Interstitial Lung Disease
MAPK	Mitogen-Activated Protein Kinase
MEK	Mitogen-Activated Protein Kinase Kinase
MSI-H	Microsatellite Instability-High
MSS	Microsatellite Stable
NCI-MATCH	National Cancer Institute Molecular Analysis for Therapy Choice
NGS	Next-Generation Sequencing
NRG1	Neuregulin 1
NSCLC	Non-Small-Cell Lung Cancer
NTRK	Neurotrophic Tropomyosin Receptor Kinase
ORR	Objective Response Rate
OS	Overall Survival
PARP	Poly (ADP-Ribose) Polymerase
PD-1	Programmed Cell Death Protein 1
PD-L1	Programmed Death-Ligand 1
PFS	Progression-Free Survival
POLE	DNA Polymerase Epsilon
POLD1	DNA Polymerase Delta 1
RET	Rearranged During Transfection
RNA	Ribonucleic Acid
ROS1	c-ros Oncogene 1 (Receptor Tyrosine Kinase)
SCC	Squamous Cell Carcinoma
SCLC	Small-Cell Lung Cancer
T-DXd	Fam-Trastuzumab Deruxtecan
TKI	Tyrosine Kinase Inhibitor
TMB	Tumor Mutational Burden
TMB-H	Tumor Mutational Burden-High
TRK	Tropomyosin Receptor Kinase
WHO	World Health Organization
